# The Late Mr. Arthur Reade

**Published:** 1912-02-10

**Authors:** 


### The Editor's Letter Box.

### THE LATE MR. ARTHUR READE.

*To the Editor of* THE HOSPITAL.

SIR,—May I add a little to your memoir on Mr. Arthur Reade? You hardly brought out how intimately Mr. Arthur Reade will always be associated with Charing Cross Hospital. It is difficult indeed for anyone who was attached to that institution as medical student and resident officer to imagine the place without Mr. Reade's genial countenance and ponderous figure, so intimately was he bound up with its varying fortunes and its every-day work. From 1882 to 1906 is a good slice of a man's life, and during that time the late secretary exercised considerable influence on the policy and progress of every department of this important London hospital. During his secretaryship, to take one or two of the far-reaching changes which were successfully engineered in his time, the imposing block of new wards facing King William Street was built in spite of many financial difficulties, and with the minimum of disturbance to the work of the hospital between 1902 and 1904. The laying of the foundation stone was carried out with full Masonic ceremony on June 20, 1902, by the Grand Master, H.R.H. the Duke of Connaught, for it may be mentioned that among Mr. Reade's numerous interests outside his work was that of freemasonry. In June, 1901, he took part in the consecration of the new Chère Reine Lodge, and was its first organist.

No man was a greater authority on the history of Charing Cross Hospital than Mr. Arthur Reade. He was continually busy at research on this subject, and unearthed many highly interesting records. This was, in fact, one of his dearest hobbies, and from time to time he generously contributed to the *Charing Cross Hospital Gazette* valuable articles containing historical accounts of the growth and progress of that institution. Among these articles one may mention the description of the ceremonial and procession at the laying of the foundation stone of the hospital on September 15, 1831, which also was performed with full Masonic splendour and solemnity.

Mr. Reade's recollections of the battle of Trafalgar Square on November 13, 1887, are also recorded in the pages of the Hospital Magazine, and his vivid description of that eventful day is greatly valued by old students.

When Mr. Reade eventually gave up office it was a long time before the staff and students grew accustomed to the idea that he was no longer one of the features of the place, and on his occasional visits he was always accorded the most hearty and homely of welcomes. The task of reconciling so many and at times conflicting interests was no light one, but Mr. Reade's tact and courtesy constantly proved irresistible.

To mourn his loss Mr. Reade leaves a widow and family; one of the sons was a student at Charing Cross Hospital, and held a resident appointment there before filling the responsible office he now holds at a large institution near London.

Yours faithfully,
AN OLD CROSS MAN.

[It is a matter, perhaps, for some surprise that Mr. Reade's historical articles have never been reprinted to supplement Dr. Hunter's interesting notes which were produced when the alterations to the Medical School were inaugurated last October.—ED. THE HOSPITAL.]

### A GOOD BOOK ON MASSAGE.

*To the Editor of* THE HOSPITAL.

